# Establishment of inflammation biomarkers-based nomograms to predict prognosis of advanced colorectal cancer patients based on real world data

**DOI:** 10.1371/journal.pone.0208547

**Published:** 2018-12-04

**Authors:** Guifang Guo, Xiuxing Chen, Wenzhuo He, Haohua Wang, Yixing Wang, Pili Hu, Yuming Rong, Lei Fan, Liangping Xia

**Affiliations:** 1 VIP Region, Sun Yat-sen University Cancer Center, Guangzhou, P.R. China; 2 State Key Laboratory of Oncology in South China, Sun Yat-sen University Cancer Center, Guangzhou, P.R. China; 3 Collaborative Innovation Center for Cancer Medicine, Guangzhou, P.R. China; 4 Department of Orthopedics, Third Affiliated Hospital, Sun Yat-Sen University, Guangzhou, P.R. China; University of Nebraska Medical Center, UNITED STATES

## Abstract

**Purpose:**

To establish three novel prognostic nomograms with inflammatory factors for advanced colorectal cancer (ACRC), right-sided colon cancer (RSCC) and left-sided colorectal cancer (LSCRC) according to real world data.

**Materials and methods:**

ACRC patients receiving medicine therapy from January 1^st^, 2005 to September 31^th^, 2015 in Sun Yat-sen University Cancer Center were enrolled. Inflammatory indicators such as the neutrophil-to-lymphocyte ratio (NLR), the platelet-lymphocyte ratio (PLR), carcinoembryonic antigen (CEA), carbohydrate antigen 19–9 (CA 19–9), lactate dehydrogenase (LDH) and C-reactive protein (CRP) were analyzed for establishing nomograms predicting overall survival (OS). Concordance index (C-index) determined predictive accuracy and discriminative ability.

**Results:**

Our study selected 807 ACRC patients, 29.6% RSCC and 70.4% LSCRC. Median OS was 23.36 months. Patients at lower level of NLR, PLR, CEA, CA 19–9, LDH and CRP showed longer OS (*P* < 0.001). For all patients, pathological grade (*P* = 0.018), treatments (*P* = 0.042), sidedness (*P* = 0.003), NLR (*P* < 0.001), CA 19–9 (*P* < 0.001), LDH (*P* < 0.001) and CRP (*P* = 0.0012) contributed to OS independently. For RSCC, pathological grade (*P* = 0.022), CA 19–9 (*P* < 0.001), LDH (*P* < 0.001) and CRP (*P* = 0.001) were significantly related with OS. For LSCRC patients, treatments (cetuximab vs chemotherapy: *P* = 0.008; bevacizumab vs chemotherapy: *P* = 0.166), NLR (*P* < 0.001), CA 19–9 (*P* = 0.030) and LDH (*P* < 0.001) were independent factors for OS. Final models showed acceptable internal validity with C-indexes of 0.687, 0.697 and 0.667 in all, RSCC and LSCRC patients.

**Conclusions:**

Inflammatory factors enrolled in the proposed nomograms showed accurately individualized prognostic prediction, and prognostic factors for RSCC and LSCRC were different.

## Introduction

Colorectal cancer (CRC) is the third most common cancer and fourth leading cause of cancer-related death globally according to the latest data [1]. The incidence and mortality rates have been declining for several decades because of historical changes in risk factors, the introduction and dissemination of screening tests, and improvements in treatment [2, 3]. Nonetheless, 18–22% patients are still diagnosed with distant metastasis and have the lowest 5-year survival rate (approximately 14%) compared with those who were diagnosed with localized and regional disease [4]. About 20% of patients with tumors in the colorectal region will develop metastasis during subsequent treatment. Oncologists have been committed to the diagnosis and treatment of patients with advanced colorectal cancer (ACRC) for decades and still must solve and overcome many obstacles. Although some patients might have the same TNM stage, the prognosis of these patients shows large heterogeneity. Nomograms for patient survival are helpful to judge the individual prognosis and develop personal management strategies. Few prognostic models including those had been established for ACRC survival although many oncologists reported the inflammatory conditions and immune status of the patients also played significant roles in cancer progression [5, 6].

Widely recognized is that inflammation strongly promotes tumor genesis, formation, angiogenesis and metastasis [5, 7]. Inflammatory factors had been reported recently to be closely related to a patient’s prognosis in colorectal carcinoma [8–10]. These factors include lactate dehydrogenase (LDH) levels, C-reactive protein (CRP) levels, the neutrophil-to-lymphocyte ratio (NLR) and the platelet-lymphocyte ratio (PLR), and the tumor biomarkers in CRC, carcinoembryonic antigen (CEA) and carbohydrate antigen 19–9 (CA 19–9), also rise under inflammatory conditions [11, 12]. These factors not only increase as inflammation appears but also as the tumor forms. Simple and regular access to detect these factors in the blood and a low cost make their use convenient and worthwhile in clinical practice.

Nomograms have been accepted as reliable tools to quantify risk by incorporating and illustrating important factors for oncologic prognosis [13]. By creating a graphic calculating scale of a statistical predictive model, a nomogram is typically used to predict the numerical probability of a clinically individualized outcome, such as overall survival (OS). Recently, several nomograms concerning the treatment of cancers, including lung cancers [14], gastric cancers [15, 16], liver cancers [17, 18], and CRCs [19, 20], have been reported. Until now, a nomogram for ACRC including all the inflammatory related factors referred in the above paragraph has not been studied. Therefore, this research established such new nomograms for ACRC, right-sided colon cancer (RSCC) and left-sided colorectal cancer (LSCRC) separately by selecting ACRC patients with palliative medicine treatment at our institution and collecting patient-related variables, tumor-dependent characteristics, histological features, treatment regimen and inflammatory factors.

## Materials and methods

### Study design and population

This study was performed at our institution to establish a prognostic model incorporating inflammatory factors and clinicopathological characteristics for predicting the prognosis of ACRC patients. Inclusion criteria: 1) older than 18 years old; 2) patients, with unresectable metastasis at the time of first diagnosis or developing ACRC after radical treatment, being treated by palliative chemotherapy with or without target therapy; 3) patients whose diagnostic time of ACRC ranged from January 1^st^, 2005 to September 31^th^, 2015; 4) all patients received at least one cycle medicine treatment after the diagnosis of ACRC; 5) all the values of inflammation-based factors concerned in our study before palliative chemotherapy could be obtained. To avoid the interference from confounding factors, we set out the exclusion criteria: 1) younger than 18; 2) patients whose data were insufficient; 3) patients who had a second tumor; 4) patients who had chronic or acute inflammatory disease; 5) patients who were receiving hormone therapy. Written informed consent was obtained for this retrospective study and patient information was anonymized and de-identified prior to analysis. The study was approved by the institutional review board and ethics committee at the Sun Yat-sen University Cancer Center. Consent was not obtained because all the data were analyzed anonymously. And the authenticity of this article has been validated by uploading the key raw data onto the Research Data Deposit public platform (www.researchdata.org.cn), with the approval RDD number as RDDA2018000867.

### Variables

To elaborate this prognostic nomogram, the following clinicopathological variables proven to predict survival in at least one previous publication were considered. Patient basic characteristics such as age, gender, and tumor-dependent characteristics such as primary tumor sidedness, and sites of metastases, as well as histological features such as pathological grade and treatment regimen, were all included in our analysis. The sites of metastasis were divided into solitary lung metastases vs the others and single liver metastases vs the others, since the patients with metastasis confined to lung had better prognosis than the others, and liver metastases were common in ACRC. TNM stage was not included in our analyses because patients enrolled in this study were all at an advanced stage. As the cases at pathological grade 1 only accounted for 4.21%, we merged it together with grade 2 as one group and grade 3 as opposite group for the following analysis. Primary tumor sidedness was divided into right-sidedness and left-sidedness according to the splenic flexure. Primary tumors in the cecum to transverse colon were coded as right-sided, and from the splenic flexure to rectum as left-sided. We focused on the inflammatory factors (LDH, CRP, PLR and NLR), and tumor biomarkers (CEA and CA 19–9). These factors were all measured at our institution before the initial chemotherapy treatments. CEA and CA 19–9 levels were regularly detected by tumor marker testing at our institution using the Roche Elecsys 2010 Chemistry Analyzer (Basel, Switzerland). LDH and CRP levels were measured in biochemical tests using the Hitachi Automatic Analyzer 7600–020 (Tokyo, Japan). The PLR and NLR, as counting values from platelets, neutrophils and lymphocytes, were measured in routine blood tests using the XE-5000TM Automated Hematology System. The normal ranges were 0–5 ng/ml, 0–35 U/ml, 109–250 U/l, and 0–8.2 mg/ l for CEA, CA 19–9, LDH and CRP, respectively. The PLR and NLR had no standard normal range as counting values. Laboratory variables were dichotomized with the cutoff at the upper limit of its normal range. In this study, we defined the cut-off of the PLR and NLR as 169 and 3, respectively, according to former published studies [21, 22].

Our primary endpoint was OS, defined as the survival time from the date of the first cycle of front-line chemotherapy treatment to the date of death from any cause. There was no secondary endpoint for this study. All the information gathered and updated from the medical records at our cancer center was permitted by our institution ethics committee.

### Statistical considerations

We first developed the prognostic model with univariate evaluating the significance of each contained clinicopathological characteristic. Next, multivariate analyses were performed using the Cox proportional hazards model, while the hazard ratios were calculated from the model coefficients. Univariate predictive variables with P < 0.05 were applied to multivariate analyses to determine the independent prognostic factors. The final Cox proportional hazards model was screened and determined by a backward step-down method based on the Akaike information criterion (AIC) as a stopping rule. One thousand boot-strap replications were used as internal validation subsets to evaluate the discrimination ability presented as the concordance index (C-index). The C-index is a generalization of the area under the curve of the receiver operating characteristic curve and can be applied to continuous outcome and censored data. Regularly, the maximum value of the c-index is 1.0, indicating perfect discrimination, whereas 0.5 indicates a random chance to correctly discriminate the outcome with the model. Nomogram and calibration plots were constructed using the rms package of R software, version 3.3.3, and all the other statistical tests were performed using SPSS 20.0. Statistical significance was set at 0.05.

## Results

### Baseline characteristics

After screening and selecting, 807 patients who met the eligibility criteria were enrolled. The main characteristics are presented in Table 1. The median age was 55 years (range 21 to 89 years). RSCC was diagnosed in 29.6% of the total cases, and LSCRC possessed 70.4%. The median OS was 23.36 months (95% CI, 21.51–25.21) for all patients, 20.11 months (95% CI, 17.93–22.28) and 24.74 months (95% CI, 22.32–27.16) for RSCC and LSCRC, respectively. For all, RSCC and LSCRC patients, the 1-year survival rates were 73.1%, 63.1% and 77.2%, respectively; the 3-year survival rates were 17.5%, 12.7% and 19.4%, respectively; and the 5-year survival rates were 4.3%, 2.5% and 5.1%, respectively. The cases with higher level values of the NLR, the PLR, LDH, CRP, CEA and CA 19–9 accounted for 37.1%, 25.8%, 29.0%, 60.5%, 71.9% and 49.4%, respectively.

**Table 1:** Baseline patient characteristics and overall survival in different groups.

		Colorectal cancer				Right-sided colorectal cancer				Left-sided colorectal cancer			
Factors		Patient number	median survial	95% CI	P-value^a^	Patient number	Median survival	95% CI	P-value^a^	Patient number	Median survival	95% CI	P-value^a^
Gender	Male	542	22.97	20.86–25.07	0.710	141	19.19	15.99–22.39	0.562	401	24.28	21.18–27.38	0.834
	Female	265	23.72	20.67–26.77		95	21.22	18.53–23.92		170	25.76	21.67–29.85	
Age	< 65	617	24.00	21.53–26.47	0.036	179	20.7	18.47–22.93	0.226	438	26.48	23.77–29.19	0.083
	≥ 65	190	20.60	17.76–23.44		57	17.74	13.73–21.75		133	22.24	19.27–25.22	
Lung metastasis	Only lung	73	32.85	26.77–38.94	0.001	17	40.97	19.02–62.92	0.037	56	32.62	25.35–39.90	0.015
	Non-pulmonary	734	22.24	20.67–23.81		219	19.19	16.65–21.72		515	23.75	21.41–26.10	
Liver metastasis	Only liver	319	20.53	18.93–22.13	0.115	86	17.74	14.57–20.92	0.150	338	22.24	19.75–24.74	0.068
	Non-hepatic	488	25.59	23.11–28.08		150	21.19	17.40–24.98		233	27.66	24.55–30.77	
Sidedness	Right	236	20.07	17.91–22.24	0.003								
	Left	571	24.61	22.23–26.99									
Pathological grade	Grade 1	34	30.55	3.53–57.58	0.058	9	30.55	1.20–59.91	0.132	25	20.07	1.81–38.34	0.238
	Grade 2	525	24.54	22.13–26.95	0.037^b^	126	25.27	22.74–27.79	0.045^b^	399	21.29	17.96–24.62	0.345^b^
	Grade 3	248	19.52	17.41–21.62		101	21.52	17.81–25.33		147	17.74	14.63–20.86	
Treatment	Chemotherapy	537	22.51	20.61–24.47	0.154	154	20.44	17.95–22.92	0.952	383	23.46	20.76–26.16	0.073
	Cetuximab	108	27.53	22.39–32.68	0.055^c^	31	21.72	18.36–25.08	0.913^c^	77	29.08	23.39–34.77	0.022^c^
	Bevacizumab	162	23.72	18.74–28.70		51	17.02	14.41–19.63		111	28.12	22.67–33.57	
NLR	≤ 3	508	28.09	25.51–30.67	<0.001	144	22.83	18.90–26.77	<0.001	364	29.5	26.71–32.30	<0.001
	> 3	299	18.23	15.93–20.54		92	13.41	11.43–15.38		207	19.65	17.35–21.97	
PLR	≤ 169	438	26.65	23.88–29.41	<0.001	107	22.97	19.95–25.98	0.007	331	28.52	25.61–31.43	<0.001
	> 169	369	19.25	17.52–20.99		129	16.56	13.93–19.18		240	20.53	17.86–23.21	
CEA	≤ 5 ng/mL	227	28.95	24.01–33.88	<0.001	66	25.59	17.63–33.56	0.003	161	30.16	24.96–35.36	0.001
	> 5 ng/mL	580	21.22	19.60–22.85		170	19.19	16.47–21.90		410	22.5	20.16–24.84	
CA19-9	≤ 37 U/mL	408	29.31	26.08–32.53	<0.001	110	27.43	21.20–33.66	<0.001	298	31.24	27.57–34.92	<0.001
	> 37 U/mL	399	19.29	17.68–20.89		126	15.67	12.38–18.96		273	20.53	18.57–22.50	
LDH	≤ 250 U/L	573	28.91	26.32–31.50	<0.001	174	21.72	18.09–25.35	<0.001	399	30.55	27.83–33.28	<0.001
	> 250 U/L	234	14.98	13.38–16.59		62	12.58	9.91–15.25		172	15.93	14.23–17.63	
CRP	≤ 3 mg/L	319	29.01	26.10–31.92	<0.001	84	27.43	23.69–31.18	<0.001	235	30.16	26.33–34.00	<0.001
	> 3 mg/L	488	19.55	18.00–21.10		152	15.93	12.38–19.49		336	21.22	19.07–23.38	

^a^ P-values were calculated using the Kaplan–Meier method with log-rank test.

^b^ P-value between grade 1–2 differentiation and grade 3 differentiation regarding OS.

^c^ P-value between target therapy (bevacizumab and cetuximab) plus chemotherapy and chemotherapy regarding OS.

NLR, the neutrophil-to-lymphocyte ratio; PLR, the platelet-lymphocyte ratio; CEA, carcinoembryonic antigen; CA 19–9, carbohydrate antigen 19–9; LDH, lactate dehydrogenase; CRP, C-reactive protein; 95% CI, 95% confidence interval.

### Univariable analyses

**Fig 1:**
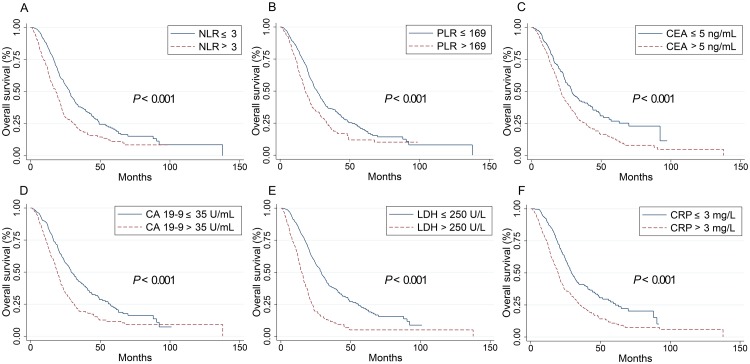
Kaplan–Meier curves for overall survival stratified by inflammation-based markers in all patients. Kaplan–Meier curves for overall survival stratified by NLR levels (A), PLR (B), CEA levels (C), CA 19–9 levels (D), LDH levels (E) and CRP levels (F). PLR: the platelet-lymphocyte ratio; NLR, the neutrophil-to-lymphocyte ratio; CEA, carcinoembryonic antigen; CA 19–9, carbohydrate antigen 19–9; LDH, lactate dehydrogenase; CRP, C-reactive protein.

**Fig 2:**
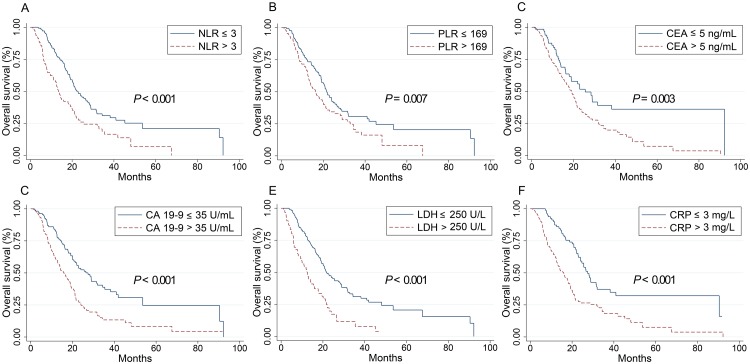
Kaplan–Meier curves for overall survival stratified by inflammation-based markers in RSCC patients. Kaplan–Meier curves for overall survival stratified by NLR levels (A), PLR (B), CEA levels (C), CA 19–9 levels (D), LDH levels (E) and CRP levels (F). RSCC, right-sided colon cancer; PLR, the platelet-lymphocyte ratio; NLR, the neutrophil-to-lymphocyte ratio; CEA, carcinoembryonic antigen; CA 19–9, carbohydrate antigen 19–9; LDH, lactate dehydrogenase; CRP, C-reactive protein.

**Fig 3:**
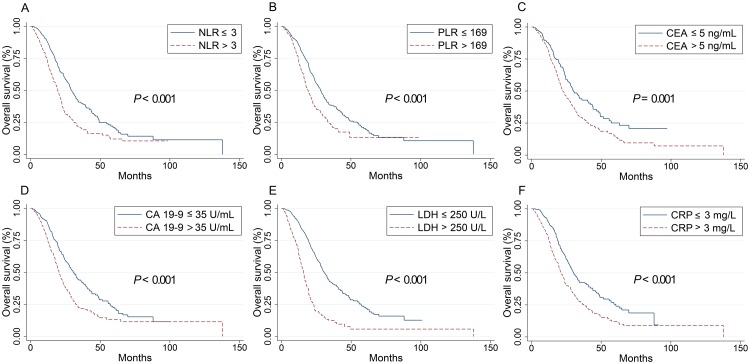
Kaplan–Meier curves for overall survival stratified by inflammation-based markers in LSCRC patients. Kaplan–Meier curves for overall survival stratified by NLR levels (A), PLR (B), CEA levels (C), CA 19–9 levels (D), LDH levels (E) and CRP levels (F). LSCRC, left-sided colorectal cancer; PLR, the platelet-lymphocyte ratio; NLR, the neutrophil-to-lymphocyte ratio; CEA, carcinoembryonic antigen; CA 19–9, carbohydrate antigen 19–9; LDH, lactate dehydrogenase; CRP, C-reactive protein.

Baseline values of all inflammatory indicators considered (NLR, PLR, CEA, CA 19–9, LDH and CRP) in this study were significantly associated with OS in the groups of all patients (P < 0.001), RSCC (P < 0.05) and LSCRC patients (P < 0.05) and higher level of these values suggested worse OS, as shown in Table 1 and Figs 1, 2 and 3. In all ACRC patients, synchronous metastasis (P < 0.001), old age (P = 0.036) and right-sided (P = 0.003), pathological grade 3 (P = 0.037) and non-solitary-lung metastases (P = 0.001) were significantly related with worse OS. Treatments (chemotherapy versus chemotherapy plus target agent; P = 0.055) exhibit a trend to impaired OS. In 527 LSCRC patients, the group who had non solitary-lung metastases (P = 0.015) or were treated with chemotherapy alone (P = 0.022) had impaired OS. In 256 RSCC patients, decreased OS were found in pathological grade 3 group (P = 0.045) and non solitary-lung metastases group (P = 0.037). However, our study found no difference in the OS between the groups with chemotherapy alone and the addition of cetuximab or bevacizumab in RSCC patients. Gender did not contribute to OS in all the three groups.

### Multivariable analyses

After univariate analysis, multivariate survival analysis using Cox’s regression model was fitted on the training subset. In all patients, pathological grade (grade 3 vs grade 1–2: HR, 1.26; P = 0.018), treatment regimens (chemotherapy with or without target agent; HR, 0.82; P = 0.042), sidedness (HR, 0.75; P = 0.003), NLR value (HR, 1.44; P < 0.001), CA 19–9 level (HR, 1.43; P < 0.001), LDH level (HR, 2.09; P < 0.001) and CRP level (HR, 1.29; P = 0.012) were associated with OS significantly. In LSCRC patients, treatment regimens (Cetuximab plus chemotherapy vs chemotherapy: HR, 0.64; 95% CI, 0.46–0.89; P = 0.008; Bevacizumab plus chemotherapy vs chemotherapy: HR, 0.81; 95% CI, 0.61–1.09; P = 0.166), NLR level (HR, 1.53; 95% CI, 1.23–1.90; P< 0.001), CA 19–9 level (HR, 1.28; 95% CI, 1.03–1.61; P = 0.030) and LDH level (HR, 2.48; 95% CI, 1.95–3.15; P < 0.001) were correlated with OS independently. In RSCC patients, pathological grade (grade 3 vs grade 1–2: HR, 1.47; 95% CI, 1.06–2.05; P = 0.022), CA 19–9 level (HR, 1.96; 95% CI, 1.41–2.73; P < 0.001), LDH level (HR, 2.04; 95% CI, 1.42–2.94; P < 0.001) and CRP level (HR, 1.83; 95% CI, 1.28–2.62; P = 0.001) were related to OS finally. All values that retained statistically relevance to OS were applied to establish the nomograms for the groups of all patients, RSCC patients and LSCRC patients. The final multivariable models retaining statistically and clinically relevant characteristics were showed in Table 2.

**Table 2:** Stratified COX proportional hazards model for overall survival.

	Colorectal cancer			Right-sided colorectal cancer			Left-sided colorectal cancer		
	HR	95% CI	P-value^a^	HR	95% CI	P-value^a^	HR	95% CI	P-value^a^
Pathological grade (grade 1–2 (reference^b^))	1.26	1.04–1.52	0.018	1.47	1.06–2.05	0.022			
Treatment									
Chemotherapy only	Reference^b^						Reference^b^		
Cetuximab plus chemotherapy	0.82	0.68–0.99	0.042				0.64	0.46–0.89	0.008
Bevacizumab plus chemotherapy							0.81	0.61–1.09	0.166
Sidedness (RSCC (reference^b^))	0.75	0.62–0.91	0.003						
NLR (≤ 3 (reference^b^))	1.44	1.20–1.73	<0.001				1.53	1.23–1.90	<0.001
CA199 (≤ 37 U/ml (reference^b^))	1.43	1.19–1.72	<0.001	1.96	1.41–2.73	<0.001	1.28	1.03–1.61	0.030
LDH (≤ 250 U/l (reference^b^))	2.09	1.70–2.57	<0.001	2.04	1.42–2.94	<0.001	2.48	1.95–3.15	<0.001
CRP (≤ 3 mg/l (reference^b^))	1.29	1.06–1.58	0.012	1.83	1.28–2.62	0.001			

^a^ P-values were calculated using the Cox-proportional hazard model.

^b^ Defining this group as reference to calculate HR value.

HR, hazards ratio; 95% CI, 95% confidence interval; NLR, the neutrophil-to-lymphocyte ratio; PLR, the platelet-lymphocyte ratio; CEA, carcinoembryonic antigen; CA 19–9, carbohydrate antigen 19–9; LDH, lactate dehydrogenase; CRP, C-reactive protein.

### Nomograms for OS and internal validation

**Fig 4:**
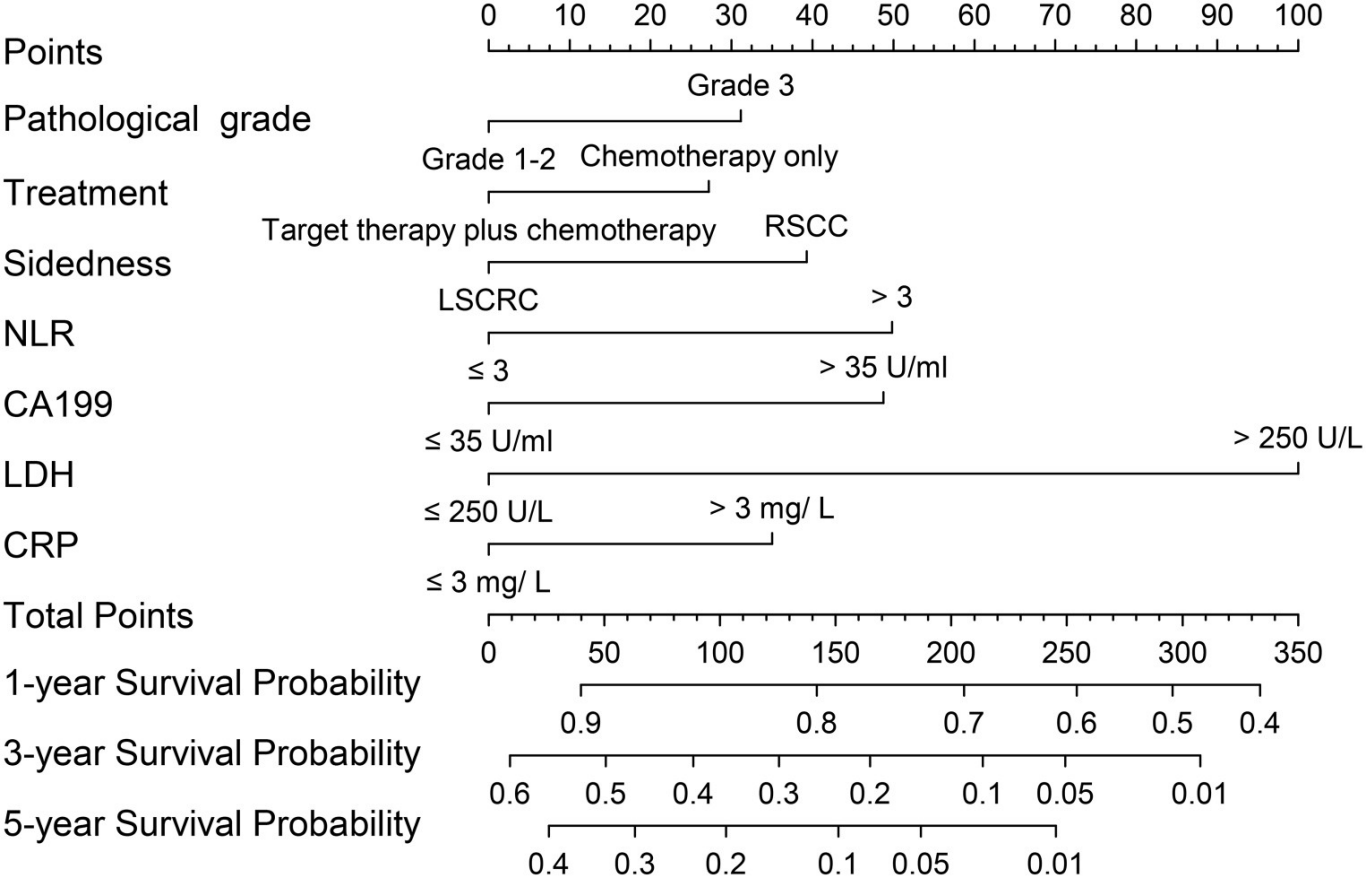
Nomogram model for all ACRC patients, including pathological grade, treatment, sidedness, NLR levels, LDH levels, CRP levels and CA 19–9 levels for 1-, 3- and 5-year OS. The user can obtain the probability of 1-, 3- and 5-year OS corresponding to a patient’s combination of covariates from the nomogram. As an example, locate the patient’s pathological grade and draw a straight line upward to the “Points” axis to determine the score related to that pathological grade. Repeat the process for each variable, sum the scores achieved for each covariate, and locate this sum on the “Total Points” axis. Draw a straight line down to determine the likelihood of the 1-, 3- and 5-year survival. ACRC, advanced colorectal cancer; OS, overall survival; NLR, the neutrophil-to-lymphocyte ratio; CA 19–9, carbohydrate antigen 19–9; LDH, lactate dehydrogenase; CRP, C-reactive protein.

**Fig 5:**
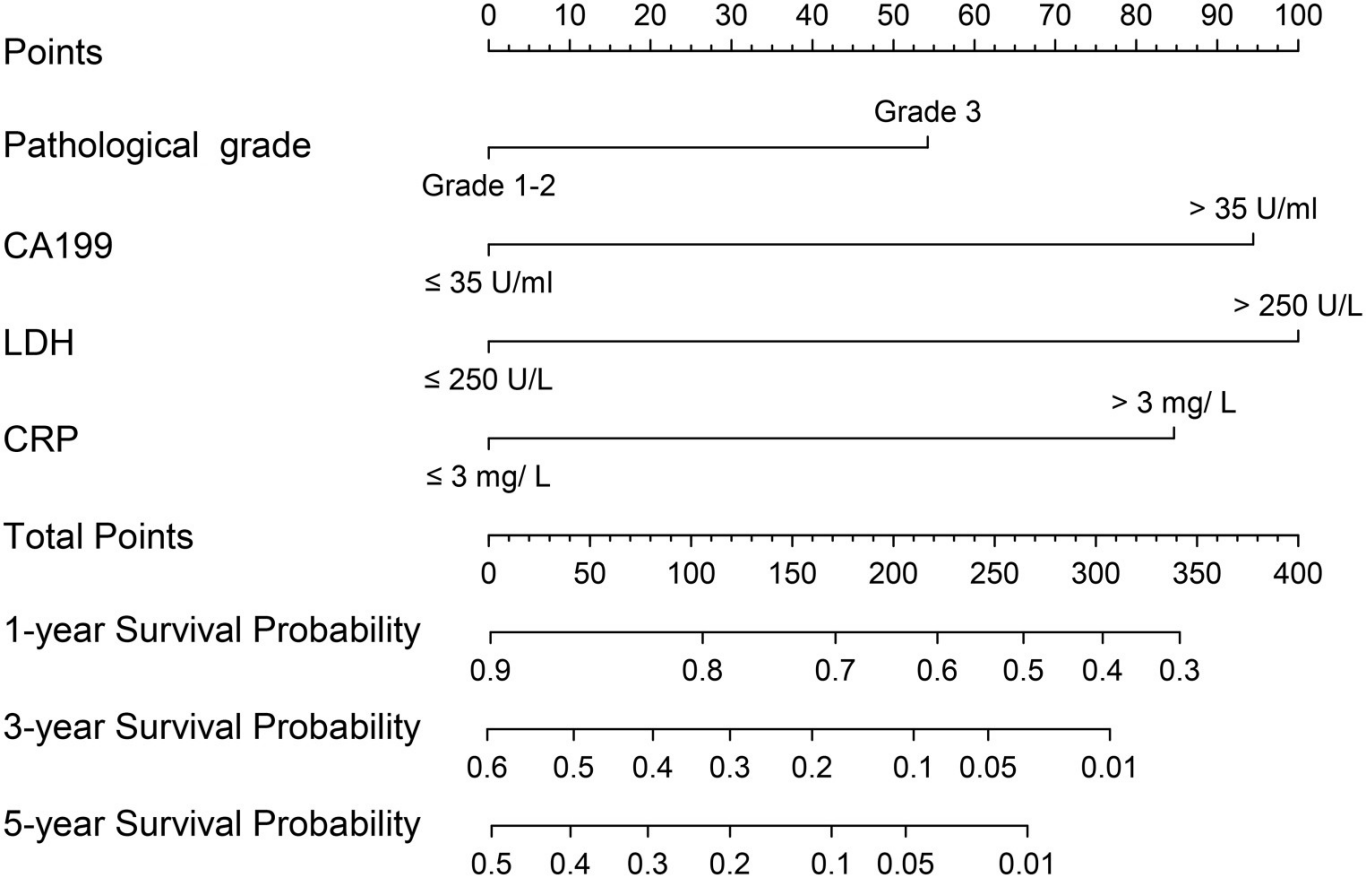
Nomogram model for RSCC patients, including pathological grade, LDH levels, CRP levels and CA 19–9 levels for 1-, 3- and 5-year OS. RSCC, right-sided colon cancer; OS, overall survival; CA 19–9, carbohydrate antigen 19–9; LDH, lactate dehydrogenase; CRP, C-reactive protein.

**Fig 6:**
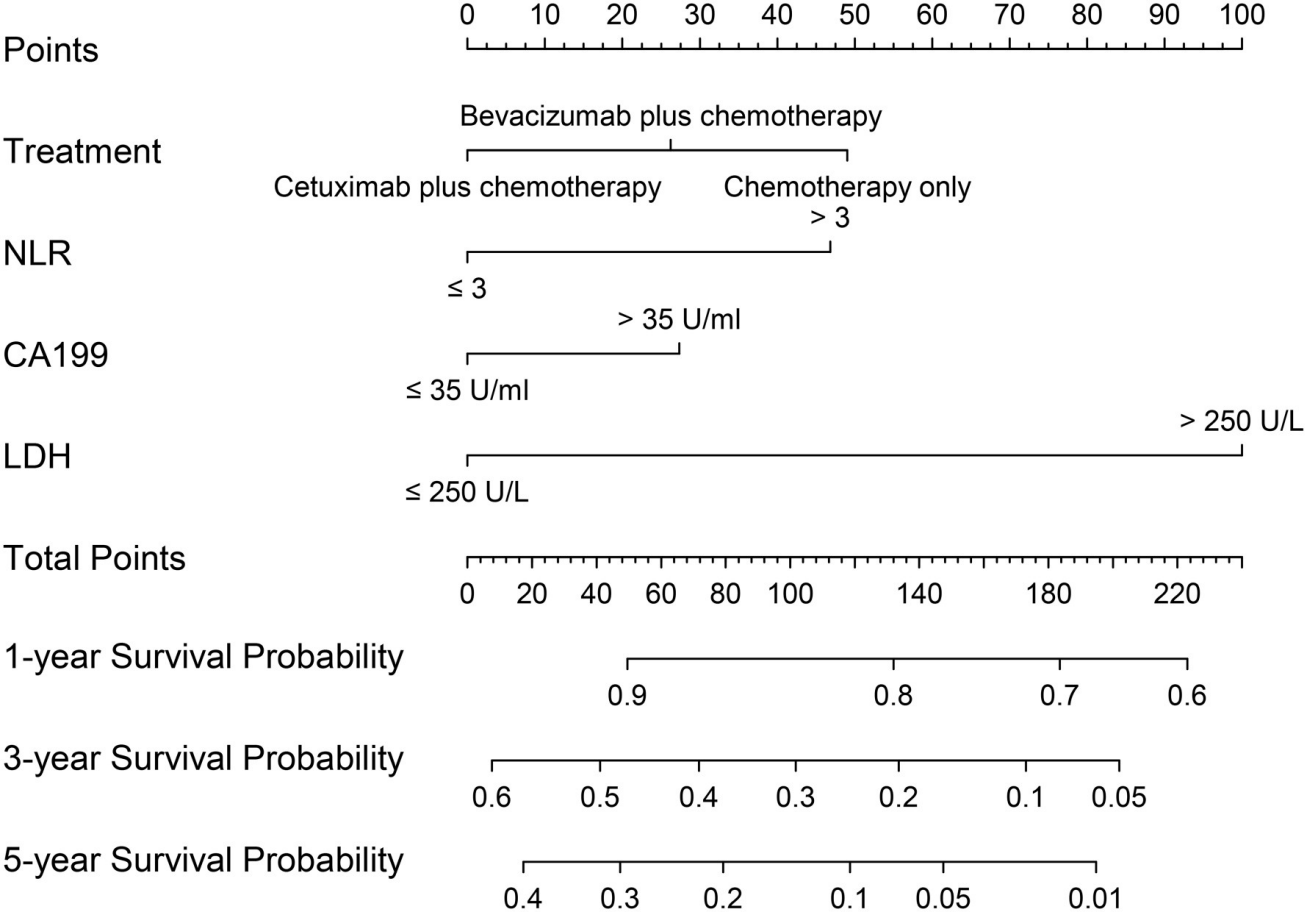
Nomogram model for LSCRC patients, including treatment, NLR values, LDH levels, and CA 19–9 levels, for 1-, 3- and 5-year OS. LSCRC, left-sided colorectal cancer; OS, overall survival; NLR, the neutrophil-to-lymphocyte ratio; CA 19–9, carbohydrate antigen 19–9; LDH, lactate dehydrogenase.

**Fig 7:**
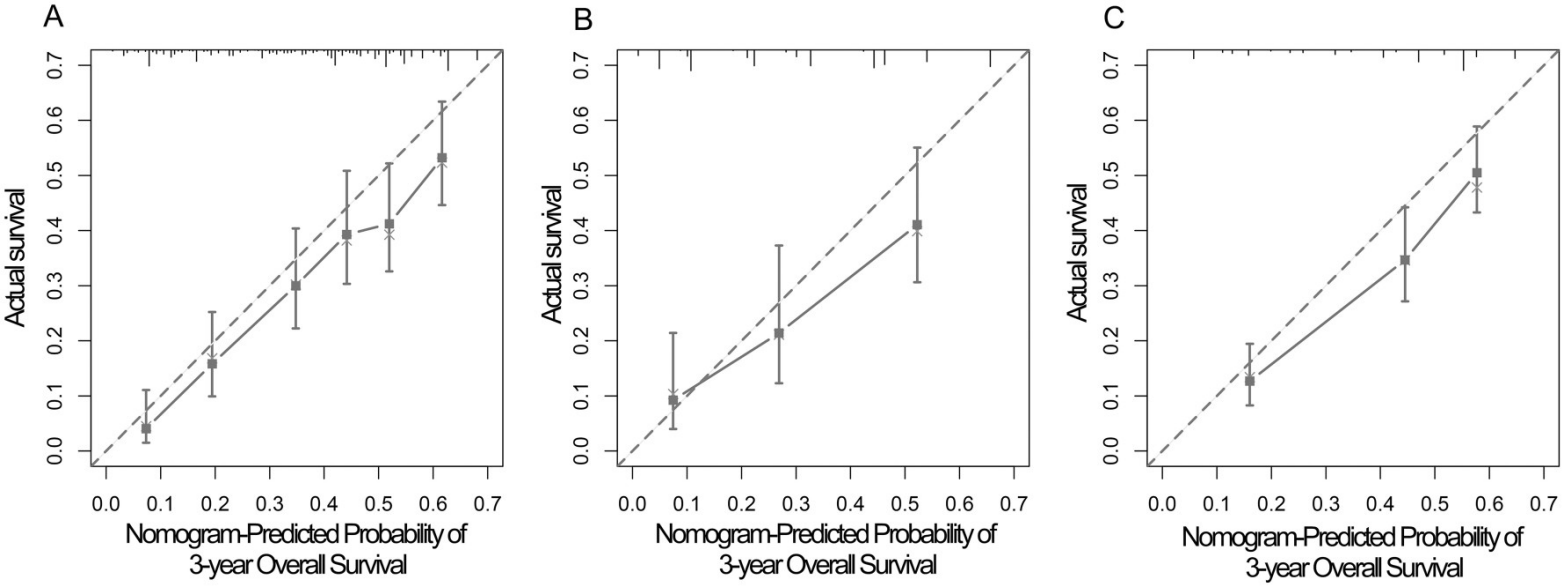
Calibration curves of the nomogram to predict OS at 3-year in all ACRC patients (A), RSCC patients (B), LSCRC patients (C). The actual OS is plotted on the y-axis; the nomogram predicted probability of OS is plotted on the x-axis. A plot along the 45-degree line would indicate a perfect calibration model in which the predicted probabilities are identical to the actual outcomes. ACRC, advanced colorectal cancer; RSCC, right-sided colon cancer; LSCRC, left-sided colorectal cancer; OS, overall survival.

The nomograms for all patients, RSCC patients and LSCRC patients are presented in Figs 4, 5 and 6 respectively, from which the probabilities of the 1-, 3- and 5-year survival rates could be predicted for every patient. In the nomogram model for total patients, high-level LDH scored the highest (100 points), followed by high-level NLR (50 points) and high-level CA 19–9 (48 points). Other factors scored less than 40 points, including right-sidedness (39 points), high-level CRP (35 points), grade 3 differentiation (31.5 points) and chemotherapy without target agent (27.5 points). In the nomogram model for RSCC, high-level LDH still scored the highest (100 points), followed by high-level CA 19–9 (95 points), high-level CRP (85 points), and grade 3 differentiation (54 points). In the nomogram model for LSCRC, high-level LDH still obtained the highest score (100 points), followed by high-level NLR (47 points), and high-level CA 19–9 (27.5 points), and the different treatment regimens obtained different points: chemotherapy without target agent (49 points) vs chemotherapy plus bevacizumab (26 points) vs chemotherapy plus cetuximab (0 points). The final multivariate models for OS showed strong internal validity with discrimination concordance index values of 0.687 (95% CI, 0.663–0.712), 0.697 (95% CI, 0.654–0.740) and 0.667 (95% CI, 0.638–0.696) for all patients, RSCC patients and LSCRC patients, respectively, indicating good calibration of the observed versus predicted outcomes as shown in Fig 7.

## Discussions

In the nomogram model of 807 patients with ACRC from our institution, we found several inflammation-related factors (LDH, NLR, CRP and CA19-9) that affected OS independently after adjusting clinicopathological variables. Grade 3 differentiation, chemotherapy without target agent, right-sidedness, increased NLR, increased CA 19–9, increased LDH and increased CRP were associated with a decreased likelihood of 1-, 3- and 5-year survival, whereas metastasis sites (solitary lung metastases vs the others, and single liver metastases vs the others), PLR and CEA did not play a prognostic role in our study. In RSCC, the worse prognostic factors were grade 3 differentiation, increased CA 19–9, increased LDH and increased CRP. In the LSCRC, chemotherapy without target agent, increased NLR, increased CA 19–9 and increased LDH were related to a worse 1-, 3- and 5-year survival rate. We established and validated three nomograms to aid the judgment of clinical prognostication and to facilitate individualized evaluation of survival rates at 1, 3 and 5 years for patients with ACRC. Our nomogram, with concordance index values of 0.687, 0.697 and 0.667 for all cases, RSCC cases and LSCRC cases, respectively, would be useful to distinguish among the patient prognoses. In addition, patients identified to be associated with low or high survival rate could be managed with more- or less-intensive therapy, and then longer OS might be promising.

Although most variables play different values in different nomogram models in our study, high-level LDH obtained the highest score (100 points) in three models. According to preclinical data, serum LDH levels were also an indirect marker of tumor hypoxia and neoangiogenesis, and clinical experience had suggested its prognostic impact [23, 24]. Several studies published recently showed LDH could be the biomarker of efficacy of anti-angiogenesis drugs in CRC, such as bevacizumab [23, 25]. As a classic tumor marker for CRC, high-level CA 19–9 scored 48, 95 and 27 in the nomogram for all patients, RSCC patients and LSCRC patients, respectively, demonstrating that CA 19–9 affected the ACRC patient prognosis regardless of sidedness, and the tightest role in RSCC. Grade 3 pathological differentiation scored 31.5 points and 54 points in the nomogram for all patients and right-sided patients, respectively, illustrating that pathological grade affected OS more in RSCC. High-level NLR obtained 50 points in the nomogram for all patients, and 47 points in LSCRC. High-level CRP scored 35 points and 85 points in the nomogram for all patients and RSCC patients, respectively. These results indicated that inflammation was closely related to the survival of ACRC. Different prognostic roles of CRP and NLR in RSCC patients and LSCRC patients indicated that different OS rates between left- and right-sidedness warranted further study. The underlying molecular mechanism will surely be explored in the future.

LSCRC patients had a longer survival time than that of RSCC patients (24.61 vs 20.07 months, respectively; P = 0.003), consistent with the latest data demonstrated by the CALGB/SWOG 80405 and FIRE3 studies [26, 27]. Treatment plan in different sidedness showed a strong correlation with ACRC patients’ prognosis in this study. For LSCRC, we found patients had a significant longer OS when receiving target agents plus chemotherapy than chemotherapy alone (P = 0.022), and patients with cetuximab addition had superior OS because cetuximab exhibited 0 score for OS in the nomogram, bevacizumab showed 26 score, and chemotherapy alone showed 49 score. By contrast, the OS among the three different treatment regimens showed no statistical significance for the patients with a right-sided primary tumor. This outcome suggested that the treatment regimen played a more critical role in the prognosis of LSCRC. Oncologists have shown worldwide consistency that cetuximab shows better OS and progression-free survival (PFS) than bevacizumab in LSCRC harboring wild-type RAS. Which treatment is better for RSCC remains controversial. In FIRE3 study, oncologists tended to administer cetuximab when the purpose of anti-tumor therapy was early tumor shrinkage. When aiming to prolong OS and PFS, patients could be given bevacizumab plus standard chemotherapy [27]. Otherwise, some studies had shown RSCC could show a better objective response rate (ORR) when receiving cetuximab in RSCC [28]. The subgroup analysis in the Chinese TAILOR study showed right-sided patients with cetuximab addition had improved ORR, PFS and OS than chemotherapy alone, while all P values were greater than 0.05. Our study found no difference in the OS between the groups with chemotherapy alone and the addition of cetuximab or bevacizumab. However, subgroup analysis in the CALGB/SWOG 80405 studies found the superiority of bevacizumab in RSCC. Until now, no prospective clinical trial comparing the different effects of cetuximab and bevacizumab on two sides is available. We must be cautious to accept the results of subgroup analysis of prospective analysis in clinical studies. In summary, two target agents added to first-line chemotherapy showed different superiority in left- or right-sided CRC based on retrospective subgroup analysis of the clinical trials.

CEA is the most famous tumor marker related to CRC prediction, prognostication, efficacy of therapy and recurrence [12, 29, 30]. We also found a close relationship between CEA and OS in ACRC (P<0.001). However, CEA failed to play a role in all three nomograms. Several reasons could account for this situation. First, only pretreatment CEA values were collected in the study. A dynamic change in the CEA level after treatment showed a closer connection with the CRC patient prognosis [31, 32]. Second, the specificity and sensitivity of CEA for CRC prognosis were not as high as the AFP for hepatocellular carcinoma [33], and some CRC cases didn’t accompany with high CEA or just accompanied with other tumor marker increasing, for example CA19-9. Third, some analyzed factors might decrease the prognostic role of CEA when they were considered in the nomogram.

Inflammatory indicators were included nomogram models for the prognosis of ACRC in our study and it was found they played different prognostic role in RSCC and LSCRC. However, there are still some limitations in our study. First, some variables related to prognosis could not be enrolled due to insufficient data, such as performance status, RAS and BRAF and MSI status, and number of metastasis sites, it would be better for model construction if they were included [34, 35]. Second, all the laboratory values included were only pretreatment value. The addition of consecutive examination of these data and comparison their changes before and after treatment would enhance their prognostic role in the nomogram. Third, regarding the retrospective internality, all the data were collected retrospectively, leading to unavoidable bias and confounding our results Memory bias is inevitable in the course of follow-up, leading to inaccuracies in certain data, such as date of death and date of first-line treatment. Similarly, because the establishment of the nomogram model requires complete data, cases with incomplete data will be excluded, which may lead to selection bias. Finally, we must mention that our three nomograms were only validated internally. The proposed three nomograms require external validation using the collected data from independent cohorts of other institution’s patients.

## Conclusions

In summary, we established nomogram models for ACRC, in which serum inflammatory values collaborating clinical and pathological characteristics showed a strong association with overall survival prediction based on real world data from our institution. Particularly, different inflammatory factors exhibited different prognostic role in RSCC and LSCRC. Our nomogram would be useful to afford the survival rate for individual patients at 1, 3 and 5 years and might be helpful to design personal treatment for ACRC.
